# Children under Five from Houses of Unclean Fuel Sources and Poorly Ventilated Houses Have Higher Odds of Suffering from Acute Respiratory Infection in Wolaita-Sodo, Southern Ethiopia: A Case-Control Study

**DOI:** 10.1155/2018/9320603

**Published:** 2018-03-18

**Authors:** Amha Admasie, Abera Kumie, Alemayehu Worku

**Affiliations:** ^1^School of Public Health, Wolaita-Sodo University, Wolaita-Sodo, Ethiopia; ^2^School of Public Health, Addis Ababa University, Addis Ababa, Ethiopia

## Abstract

**Background:**

Acute respiratory tract infection is the most common illness in childhood. Ninety-five percent (99% of rural and 80% of urban) of households in Ethiopia primarily use solid fuel for cooking. This study investigated the effect of household fuel use and house ventilation on acute respiratory infection in children, Wolaita-Sodo, Southern Ethiopia.

**Methods:**

A community based case-control study design was used, covering a sample of 1144 children with ratio of 1 : 3 (286 cases and 858 controls) aged between 0 and 59 months. A case was defined as a child who suffered from cough, followed by short, rapid breathing in the last two weeks that preceded the survey, while control was defined as a child who had not any of the respiratory infection signs and symptoms. Study subjects were recruited after a census from households. Data were entered using EpiData version 3.1 and analyzed using SPSS version 21.

**Results:**

The proportion of children aged 1–3 years and 3–5 years was 76% and 24%, respectively. Two-thirds of children lived in households that used solid fuels for cooking (charcoal 62.76% and biomass 24.73%). The majority of households (83%) used open/traditional three-stone stoves. Unclean fuel users for cooking (AOR = 2.09, 95% CI 1.03–4.22), poorly ventilated houses (AOR = 4.32, 95% CI 2.61–7.15), large family size (AOR = 1.85, 95% CI 1.31–2.62), and carrying of a child while cooking (AOR = 1.66, 95% CI 1.18–2.34) were significant risk factors of acute respiratory infection in children under five.

**Conclusions:**

Children from houses of unclean fuel sources and poorly ventilated houses were more likely to be affected by acute respiratory infection. Using clean energy sources and improved stoves is highly suggested.

## 1. Background

The entire world used solid fuels (biomass and coal) for cooking more than a century; now more than 60% use gas and/or electricity-fuels that are basically clean at use [1]. Households burn such solid fuels in inefficient devices and in poorly ventilated kitchens. These result in very high exposures to multiple toxic products of incomplete combustion [2–4]. People in developing countries are commonly exposed to very high levels of pollution for 3–7 hours daily over many years [5]. Most of daily exposures to household air pollution (HAP) are because of the greater amount of time spent in household and the higher pollution levels of households [6]. HAP varies depending on the type of fuel, stove, housing characteristics, fuel moisture content, the practice of the people using the stoves, and the stove technology [7]. Unclean fuels are often burned in inefficient, poorly vented combustion devices. The incomplete combustion of these solid fuels results in much of the fuel energy being emitted as potentially toxic pollutants [8]. Biomass fuel combustion results in severe household air pollution and has been associated with increased risk for acute lower respiratory tract infections [9]. The incidence of ARI was higher in children who live in houses where biomass fuel was used and who accompanied their mothers while cooking compared with children living in houses [10]. The odds of having suffered from ARI were large with woodstoves homes relative to charcoal stoves [11]. An exposure to indoor air pollution was risk factor for severe lower acute respiratory infections in children [12].

A study in Ghana indicated that 99% of the households used biomass as their primary fuel, 55% cooked outdoors, and 77% cooked on traditional fires [13]. According to 2016 Ethiopian Demographic and Health Survey (EDHS) report, 93% (70.6% of urban and 98.8% of rural) of households use solid fuel as a primarily source of energy for cooking [14]. A study in Gondar and Addis Ababa revealed that 54.7% and 60% of the children lived in households using biomass fuels, respectively; furthermore biomass fuel users were more likely to have ARI in children than clean fuel users in both studies [15, 16].

Culturally, in Ethiopia women are expected to cook foods, care for children, and serve their families. Women carry their child on their back while cooking and children spent hours close to cooking fire with their mother and consequently have high exposures to health-damaging pollutants. The housing design and structures of houses in Ethiopia are not suitable in ways that can exhaust household air pollutants; as a result, incomplete combustion of fuels and poor ventilation result in high household concentrations of health-damaging pollutants and women and young children are exposed to high level of household air pollution every day. Hence, the aim of this study was to investigate the effects of household fuel use and house ventilation on acute respiratory infection in children in Wolaita-Sodo, Southern Ethiopia.

## 2. Methods

### 2.1. Study Context

The study was conducted in Wolaita-Sodo town in February 2017. Wolaita-Sodo town is located 329 kilometers away from Addis Ababa. The town has three subcities and 11 districts with a population of 111,616 and 22,777 households. The number of women whose age is between 15 and 49 years was 3829; and the number of children under five was 17,421. The town has one private and one government hospital. There are health centers, private clinics, and drug vendors. Sources of fuels used for cooking in the households of Wolaita-Sodo town are firewood 94.6%, charcoal 96.6%, kerosene 11.5%, electricity 14.2%, crop residue 2.02%, dung cake 6%, and saw dust 23.7% [17].

### 2.2. Study Design

A community based unmatched case-control study design was used to ascertain the relationship between unclean fuel source used for cooking and acute respiratory infection in children aged between 0 and 59 months.

### 2.3. Study Population

All children under five in the selected districts were source population. The study population was the selected children under five whose age was between 0 and 59 months paired with their mother or care giver, who were living in Wolaita-Sodo town. Eligibility criteria were availability of a child to be screened for ARI, residents of Wolaita-Sodo town (living at least six months in Wolaita-Sodo town), and one child per household only. The study excluded a mother or care giver of a child who was seriously ill during data collection or a child who was on treatment for confirmed tuberculosis.

### 2.4. Selection of Cases and Controls

The presence of ARI cases in children was assessed by interviewing of their mothers or care givers using WHO criteria of ARI case definition. This is in agreement with existing literatures [18, 19]. During the census, those children who fulfilled the WHO criteria of ARI were reconfirmed by the supervisors using the same criteria. Control was defined, if a child had not any acute respiratory infection sign and symptoms.

### 2.5. Sample Size and Sampling Procedure

The sample size calculation was determined based on two-population proportion formula accounting for unmatched case-control study design. The design considered a confidence level of 95% and power of 80% and ratio of case-to-control number was 1 : 3, and the least extreme odds ratio to be detected was 2.00. A proportion of controls with biomass fuel exposure (17.4%) were taken from a study conducted in rural area of Nepal [5]. Based on the above assumptions, the sample size was calculated by the Open Epi Version 3.03 (Open source Epidemiologic Statistics for Public Health). Having considered a design effect of 2 and a 10% of nonrespondent rate, the final sample size was 1148 (i.e., 287 cases and 861 controls).

The town has three subcities, with a total of 11 districts. Six districts were selected randomly. The selected districts were clusters with assumption of homogenous characteristics. Census was conducted in the selected districts in order to enumerate the number of children, as cases and controls, code households, and setting sampling frame. Based on the census data, all sample sizes (1148 households) were allocated based on Probability Proportional to Size (PPS) of the population in each selected district. Then, each household was selected using Systematic Random Sampling (SRS) technique, at the interval of every other case child. When the case child had been once selected, then the next three consecutive control's households were interviewed. If there were two children under five in the same house, one child was selected randomly using lottery method.

### 2.6. Data Collection Procedures and Tools

Interviewer administered face-to-face interview was conducted using a close-ended structured questionnaire that contains sociodemographic variables, the exposure related questions, fuel use, stove type, housing characteristics, type of kitchen, cooking practice, smoking history (smoking status, pack-years of smoking, and second-hand smoke), occupational history, and medical history. The questionnaire was adopted from WHO and Demographic and Health Survey (DHS) questions to meet the context of the study area [20].

Data collectors were public health officers who were trained for three days on the content of the questionnaire, how to screen and refer sick children to a clinic. Each data collector did 5–7 households pretesting of the questionnaire. Based on the pretest input, the questionnaire was restructured and remodified. Two supervisors (M.S. in Public Health) were employed who took similar training to data collectors and their role was to supervise the completeness, accuracy, and consistency of data and reconfirm cases.

### 2.7. Operational Definition

*Acute Respiratory Infection (ARI)*. This includes a child who suffered from cough, followed by short, rapid breathing in the last two weeks that preceded the survey [21].

*Improved Water Source*. This includes a piped source within the dwelling, yard, or plot; a public tap/stand pipe, or borehole; a protected well; spring water and rainwater [14]. 

*Level of House Ventilation*. This includes the following: good (a house with more than one door and one window), moderate (a house with one door and one window), and poor (a house with only one door and no window).

### 2.8. Data Management and Analysis

The collected data were reviewed and checked for completeness by the supervisors and principal investigators. Completed questionnaire was coded and entered into computer software program, EpiData version 3.1. Data cleaning and analysis were done using SPSS version 21. Significantly associated variables in unconditional bivariate logistic analysis were entered into a multivariable analysis using Enter Method of Regression. Each variable was tested for collinearity (at Variance Inflation Factor < 10 and Tolerance Test > 0.1) prior to multivariate analysis. Hosmer-Lemeshow goodness-of-fit test was used in multivariable analysis to check the adequacy of the model. In both analyses, the cut-off point for *P* value was <0.05. Finally, data were presented in tables and narration.

### 2.9. Data Quality Assurance

During the census and data collection time, houses were labeled with a code number and the code was recorded on the questionnaire. Regular supervision and follow-up were made by supervisors and the principal investigator. To ensure the quality of data, high emphasis was given in designing data collection instrument. Prior to the data collection pretest was made on 60 households which accounted for 5% of the total sample size in nonselected districts. ARI cases were screened by data collectors using WHO case definition. Cases were verified by supervisors by asking similar questions to the mothers and care takers.

### 2.10. Ethical Consideration

Ethical clearance to undertake the study was obtained from Addis Ababa University, College of Health Sciences, Institutional Review Board Office. Informed consent to participate in the study was obtained before conducting the interview. For this, one-page subject information sheet and informed consent letter were attached to each questionnaire. Participation was in voluntary manner and private information was confidential. Participants were informed that participating in this research has no any risk or harm to the child as well as to the whole family too. The right of the respondent to withdraw from the interview or not to participate was respected. All children who suffered from ARI in the course of the screening period were referred to the nearby health facility for appropriate medical attention.

## 3. Results

### 3.1. Sociodemographic Characteristics

A total of 1144 children (286 cases and 858 controls) were included in the study with response rate of 99.6%. The mean age of the mother was 26.89 ± 6.0 years. Two hundred ten (18.36%) of mothers were employed, while 544 (47.55%) were housewives. Eight hundred thirty-one (72.6%) of the households had a family of 5 or less (Table 1).

### 3.2. Maternal and Child Related Variables

Children were relatively equally distributed by age. The mean age of the children was 24.15 ± 14.98 months. The largest age group of 330 (28.86%) were between 12 and 23 months. Males accounted for 606 (52.97%) of the total children. Almost half of the children, 642 (56.1%) of children, had a birth interval less than or equal to two years. Seven hundred forty-five (65.12%) of children shared their bed room with two people (Table 2).

### 3.3. Kitchen and House Characteristics

About 712 (62.23%) of households had a kitchen separated from the main house, while 351 (30.68%) of households had kitchen inside the living house. Only, 417 (58.5%) of the kitchen had no chimney; 666 (93.54%) did not open windows during the cooking time. About 508 (44.4%) of children lived in the house of one window (Table 3).

### 3.4. Type of Fuel, Stove, and Home Practice

Almost two-thirds of children live in households that used polluting fuel for cooking, charcoal 718 (62.76%), followed by biomass fuel 283 (24.73%); that is, only 143 (12.5%) of the households used clean fuel energy, such as electricity, biogas, and Liquefied Petroleum Gas (LPG). The majority, 950 (83.04%), of the households used open/traditional three-stone stoves. Five hundred fifty-two (44.75%) of the respondents spent at least 2 hours per day in cooking foods. Only one-third of the children were present around the cooking stove while mothers were cooking meal. Almost less than one-third of children had a habit of being carried by their mothers or care giver while cooking meal (Table 4).

### 3.5. Bivariate and Multivariate Analysis

Predictor variables of acute respiratory infection in children under five were analyzed using binary logistic regression, and hence significantly associated variables in bivariate analysis were entered to the multivariable logistic regression model. Unclean fuels (biomass fuel and charcoal) used for cooking showed a significant effect on children ARI as compared to unclean fuels (electricity, LPG, and biogas) (OR: 2.09; 95% CI 1.03–4.22). The odds of ARI were four times higher in children who were living in poorly ventilated houses (only one room, one door, and no window) than living in houses having good ventilation (more than two rooms, doors, and windows) (OR: 4.32; 95% CI 2.61–7.15). The odds of ARI in children were nearly twice higher in households with family members of more than five than they were in family members of five or less (OR 1.85; 95% CI 1.31–2.62). Children being carried on the back of their mother showed a more significant association than those who had never been carried (OR 1.66; 95% CI 1.18–2.34) (Table 5).

## 4. Discussion

Biomass and charcoal fuel are chief sources of fuel by almost all participant households' in this study. From the results, the main type of cooking fuel in the household was analyzed against children ARI. Unclean fuel users showed a significant effect on children ARI as compared to clean fuel users for cooking. This study result was in line with previous studies. Biomass cooking fuel was significantly associated with acute lower respiratory tract infection in India (OR 4.73, 95% CI 1.67–13.45) [9]; the incidence of ARI was higher in children living in houses where biomass fuel was used for cooking (RR 1.5, 95% CI 1.2–1.9) [10] and in a study of systematic review and meta-analysis, the overall pooled odds ratio of unprocessed fuel use and risk of pneumonia was (OR 1.78, 95% CI 1.45–2.18) [22]. Generally, children from biomass fuel user households were more likely to suffer from ARI than clean fuel users. The reason could be that biomass fuel and charcoal emit small sized particulate matter which is able to deposit in the lungs. Deposited particulate matters may alter airway reactivity to antigens or affect the ability of the lungs to handle bacteria, suggesting that exposure may result in enhanced susceptibility to microbial infection [4]. This implies that if children were exposed to biomass and charcoal fuel, they would have higher chance of getting ARI.

The number of doors and windows is pertinent in improving house ventilation and also associated with children ARI. The odds of ARI in children who are living in poorly ventilated houses were four times higher than living in houses that had good ventilation. This study was consistent with the study conducted in slum areas of Addis Ababa, with odds ratio of 2.96 (95% CI: 1.38–3.87) in the study houses [16]. This indicated that smokes from poorly ventilated houses could lead to higher chance of exposing the child.

A habit of carrying children on the back of mothers was linked to children ARI; the odds of ARI were twice higher in children who had been carried on the back while cooking than those who had never been carried. This was similar to the study done in Gondar, 2.68 (95% CI: 1.12–6.48) [15], in Addis Ababa, 1.78 (95% CI: 1.09–2.87) [16]. Both studies revealed that a child who had been carried on the back of mothers while cooking had more risk of developing ARI. Carrying child on the back of mothers while cooking is a deep rooted culture in Ethiopia, where women are responsible for taking care of their children and preparing meal to the family. In this study, women spent much time in cooking to serve the family, with the mean and standard deviation of 2.24 ± 1.06 hours, which was consistent with the study of Taylor and Nakai, where women spent 1–3 hours in the kitchen [11], and the study of Clark et al. depicted that women spent a long time in cooking activities with a mean and standard deviation of 6.5 ± 4.3 hours in a day [23]. Both studies revealed that ARI prevalence appears to increase with increase in time spent in kitchens. This implies that if children stayed on the back of their mother for longer period, child would have equal level of smoke exposure as their mothers do.

In terms of the household's family size, the odds of ARI in children were twice higher in households with a family member of more than five than they were in a family member of five or less. The result of this study was in agreement with the study done in central India, where 53.2% of cases with history of overcrowding in family had a high risk of ARI compared to the control group, 22.8% (*P* < 0.0001, OR = 3.84) [24]; in Nigeria, a family size of more than 5 siblings living in a home was shown to affect significantly the prevalence of pneumonia [25], and a systematic study with meta-analysis revealed that crowding of a house (more than 7 persons in a household or more than 2 persons per room) is a risk factor for severe acute lower respiratory infection [12]. The implication of large family sizes was that the crowding of a house affects the health of inhabitants with high chance of transmission of droplets of infective organisms between them.

## 5. Limitations of the Study

The study lacked individual exposure measurements of specific pollutants like particulate matter, carbon monoxide, and others. This research also might have suffered a recall bias as case-control studies are inherent with this type of bias. The research team tried to operationalize variables, used appropriate and professional data collectors and supervisors, and used standard method of classifications of cases and controls.

## 6. Conclusion

Unclean fuel source, primarily biomass fuel and charcoal, had an effect on acquiring of ARI. Carrying child on the back during cooking time and child living with a family of more than five were determinant factors for the development of child ARI. Therefore, the most feasible recommendation is to distribute locally accessible and affordable clean energy supply to the community, particularly improved cook stoves. The community should therefore be given health education on the preventive measures of ARI, the role of biomass fuel combustion, house ventilation, and family planning programs. Mothers should be informed about the threats of engaging and carry children on the back during cooking time. Further research is required to determine the level of HAP from biomass fuels using more direct measures of exposure.

## Figures and Tables

**Table 1:** Relationship between sociodemographic characteristics of households in ARI, Wolaita-Sodo, Ethiopia, February 2017 (*n* = 1144).

Sociodemographic characteristics	Case, *n* (%)	Control, *n* (%)	Total, *n* (%)
Mother age			
15–24 years	94 (27.2)	252 (72.8)	346 (30.24)
25–44 years	189 (24.2)	592 (75.8)	781 (68.26)
45+ years	3 (17.6)	14 (82.4)	17 (1.48)
Educational status of the mother			
No education	98 (8.6)	233 (20.4)	331 (28.9)
Primary level	109 (33.4)	217 (66.6)	326 (28.50)
Secondary level	46 (18.7)	200 (81.3)	246 (21.5)
Higher level	33 (13.7)	208 (86.3)	241 (21.07)
Employment			
Employed	30 (14.3)	180 (85.7)	210 (18.36)
Petty trade	37 (18)	168 (82)	205 (17.92)
Unemployed	61 (5.3)	124 (10.8)	185 (16.2)
Housewife	158 (29)	386 (71)	544 (47.55)
Family size			
≤5 families	190 (22.9)	641 (77.1)	831 (72.6)
>5 families	96 (30.7)	217 (69.3)	313 (27.4)
Main drinking water source			
Improved	264 (23.1)	817 (71.4)	1081 (94.5)
Nonimproved	264 (23.1)	41 (3.6)	63 (5.5)
The type of latrine			
No latrine	10 (20)	40 (80)	50 (4.37)
Pit latrine	246 (25.3)	726 (74.7)	972 (84.96)
VIPL	21 (22.1)	74 (77.9)	95 (8.3)
Water pouring	9 (33.3)	18 (66.7)	27 (2.36)

**Table 2:** Relationship between maternal and child related variables in ARI, Wolaita-Sodo, Southern Ethiopia, February 2017 (*n* = 1144).

Characteristics	Case, *n* (%)	Control, *n* (%)	Total, *n* (%)
Sex of the child			
Male	145 (23.9)	461 (76.1)	606 (53)
Female	141 (26.2)	397 (73.8)	538 (47)
Child age			
0–11 months	62 (22.1)	219 (77.9)	281 (24.6)
12–23 months	99 (30)	231 (70)	330 (28.8)
24–35 months	57 (22)	202 (78)	259 (22.6)
36–47 months	43 (25)	129 (75)	172 (15)
48–59 months	25 (24.5)	77 (75.5)	102 (8.9)
ANC follow-up			
Yes	274 (24.8)	832 (75.2)	1106 (96.68)
No	12 (31.6)	26 (68.4)	38 (3.32)
Birth interval			
>=2 years	177 (72.4)	465 (27.6)	642 (56.1)
>2 years	109 (21.7)	393 (78.3)	502 (43.9)
Child schooling			
Yes	60 (29.6)	143 (70.4)	203 (17.74)
No	226 (24)	715 (76)	941 (82.25)
Bedroom shared with the child			
1 person	48 (23.4)	157 (76.6)	205 (17.91)
2 persons	186 (25)	559 (75)	745 (65.12)
>=3 persons	52 (26.8)	142 (73.2)	194 (16.95)
Breastfeeding started time			
Within 1 hour	214 (24.8)	650 (75.2)	864 (87.71)
Within 24 hours	25 (24.8)	76 (75.2)	101 (10.25)
>24 hours	7 (35)	13 (65)	20 (2)

**Table 3:** Relationship between kitchen and house characteristics in ARI, Wolaita-Sodo, Ethiopia, February 2017 (*n* = 1144).

Kitchen and house characteristics	Case, *n* (%)	Control, *n* (%)	Total, *n* (%)
Kitchen arrangement			
Inside the house	125 (35.6)	226 (64.4)	351 (30.68)
Separate building	136 (19.1)	576 (80.9)	712 (62.23)
Outdoor/open	25 (30.9)	56 (69.1)	81 (7.08)
Separate kitchen			
Yes	136 (19.1)	576 (80.9)	712 (62.2)
No	150 (34.7)	282 (65.3)	432 (37.8)
Kitchen has a chimney			
Yes	48 (16.3)	247 (83.7)	295 (41.43)
No	88 (22.1)	329 (78.9)	417 (58.56)
Floor material of kitchen			
Soil/mud	127 (19.2)	533 (80.8)	660 (92.7)
Cement	9 (17.3)	43 (82.7)	52 (7.3)
Windows open during cooking?			
Yes	12 (26.1)	34 (73.9)	46 (6.46)
No	124 (18.4)	542 (81.4)	666 (93.54)
# of rooms			
1 room	118 (34)	229 (66)	347 (30.33)
2 rooms	106 (34)	366 (77.5)	472 (41.25)
3+ rooms	62 (19.1)	263 (80.9)	325 (28.4)
# of windows			
No window	22 (34.4)	42 (65.6)	64 (5.59)
1 window	159 (31.3)	349 (68.7)	508 (44.4)
2 windows	67 (17.1)	325 (82.9)	392 (34.26)
3+ windows	38 (21.1)	142 (78.9)	180 (15.73)

**Table 4:** Relationship between fuel types, stoves, and home practices in ARI, Wolaita-Sodo, Ethiopia, February 2017 (*n* = 1144).

Characteristics of fuel and stove types	Case, *n* (%)	Control, *n* (%)	Total, *n* (%)
Type of fuel mainly used for cooking			
Biomass fuel	143 (50.5)	140 (49.5)	283 (24.73)
Charcoal	122 (17)	596 (83)	718 (62.76)
Electricity	21 (14.7)	122 (85.3)	143 (12.5)
Type of fuel mainly used for cooking			
Clean fuel	21 (14.7)	122 (85.3)	143 (12.5)
Nonclean fuel	265 (26.5)	736 (73.5)	1001 (87.5)
Type of cooking stove usually used			
Open stove	258 (27.2)	692 (72.8)	950 (83.04)
Improved stove	11 (10.9)	90 (89.1)	101 (8.82)
Electric stove	17 (18.3)	76 (81.7)	93 (8.12)
Time spent in cooking			
1 hour	66 (24.4)	205 (75.6)	271 (23.7)
2 hours	120 (23.4)	392 (76.6)	512 (44.8)
3+ hours	100 (27.7)	261 (72.3)	361 (31.6)
Child usually present during cooking			
Yes	123 (32.1)	260 (67.9)	383 (33.47)
No	163 (21.4)	598 (78.6)	761 (66.52)
Habit of carrying a child while cooking			
Yes	106 (34.4)	202 (65.6)	308 (26.9)
No	180 (21.5)	656 (78.5)	836 (73.07)
Family member smokes cigarette			
Yes	11 (50)	11 (50)	22 (1.92)
No	275 (24.5)	847 (75.5)	1122 (98.0)

**Table 5:** Logistic regression model for ARI, Wolaita-Sodo, Southern Ethiopia, February 2017.

Predictor variables	Case	Control	AOR (95% CI)	COR (95% CI)
Educational status of the mother				
Illiterate	98	233	1.40 (1.05–1.86)^*∗*^	0.94 (0.67–1.31)
Literate	188	625	1.00	1.00
Employment status				
Employed	30	180	1.00	1.00
Petty trade	37	168	1.32 (0.78–2.24)	1.10 (0.62–1.96)
Unemployed	61	124	2.95 (1.80–4.83)^*∗*^	2.21 (1.27–3.85)^*∗*^
Housewife	158	386	2.46 (1.60–3.77)^*∗*^	1.97 (1.23–3.15)^*∗*^
Family size				
≤5 families	190	641	1.00	1.00
>5 families	96	217	1.49 (1.12–1.99)^*∗*^	1.85 (1.31–2.62)^*∗*^
Birth interval				
<=2 years	177	465	1.37 (1.04–1.80)^*∗*^	1.41 (1.03–1.92)^*∗*^
>2 years	109	393	1.00	1.00
Number of rooms in the house				
1 room	118	229	2.18 (1.53–3.11)^*∗*^	1.46 (0.81–2.66)
2 rooms	106	366	1.22 (0.86–1.74)	1.15 (0.70–1.90)
3+ rooms	62	263	1.00	1.00
Number of windows of the house				
No window	22	42	1.95 (1.04–3.66)^*∗*^	0.77 (0.32–1.84)
1 window	159	349	1.70 (1.13–2.55)^*∗*^	0.71 (0.37–1.38)
2 windows	67	325	0.77 (0.49–1.20)	0.51 (0.29–1.92)
3+ windows	38	142	1.00	1.00
Level of house ventilation				
Poor	186	288	4.62 (2.92–7.30)^*∗*^	4.32 (2.61–7.15)^*∗*^
Moderate	75	391	1.37 (0.84–2.23)	1.58 (0.93–2.68)
Good	25	179	1.00	1.00
Kitchen arrangement				
Inside the house	125	226	1.23 (0.73–2.08)	1.54 (0.85–2.76)
Separate building	136	576	0.52 (0.31–0.87)^*∗*^	0.70 (0.39–1.26)
Outdoor/open	25	56	1.00	1.00
Type of fuel mainly used for cooking				
Clean fuel	21	122	1.00	1.00
Nonclean fuel	265	736	2.09 (1.29–3.39)^*∗*^	2.09 (1.03–4.22)^*∗*^
Type of stoves usually used				
Open stove	258	692	1.00	1.00
Improved stove	11	90	0.32 (0.17–0.62)^*∗*^	0.62 (0.30–1.24)
Electric stove	17	76	0.60 (0.34–1.03)	1.91 (0.87–4.20)
Child usually present during cooking				
Yes	123	260	1.73 (1.31–2.28)^*∗*^	1.28 (0.92–1.78)
No	163	598	1.00	1.00
Habit of carrying a child while cooking				
Yes	106	202	1.91 (1.44–2.55)^*∗*^	1.66 (1.18–2.34)^*∗*^
No	180	656	1.00	1.00
Family member smoke cigarette				
Yes	11	11	3.08 (1.32–7.18)^*∗*^	1.84 (0.69–4.90)
No	275	847	1.00	1.00

*∗* indicates those that are significant at *P* value < 0.05; 1.00 indicates the reference category.
